# Supplementary material for: Trends in Systemic Inflammatory Reaction (SIR) during Paclitaxel and Carboplatin Chemotherapy in Women Suffering from Epithelial Ovarian Cancer
Source: Cancers (Basel). 2023 Jul 13;15(14):3607. doi: 10.3390/cancers15143607 (PMC10377399; doi:10.3390/cancers15143607)
Supplement: Supplementary file 1 [file cancers-15-03607-s001.zip › cancers-2296762-supplementary.pdf]

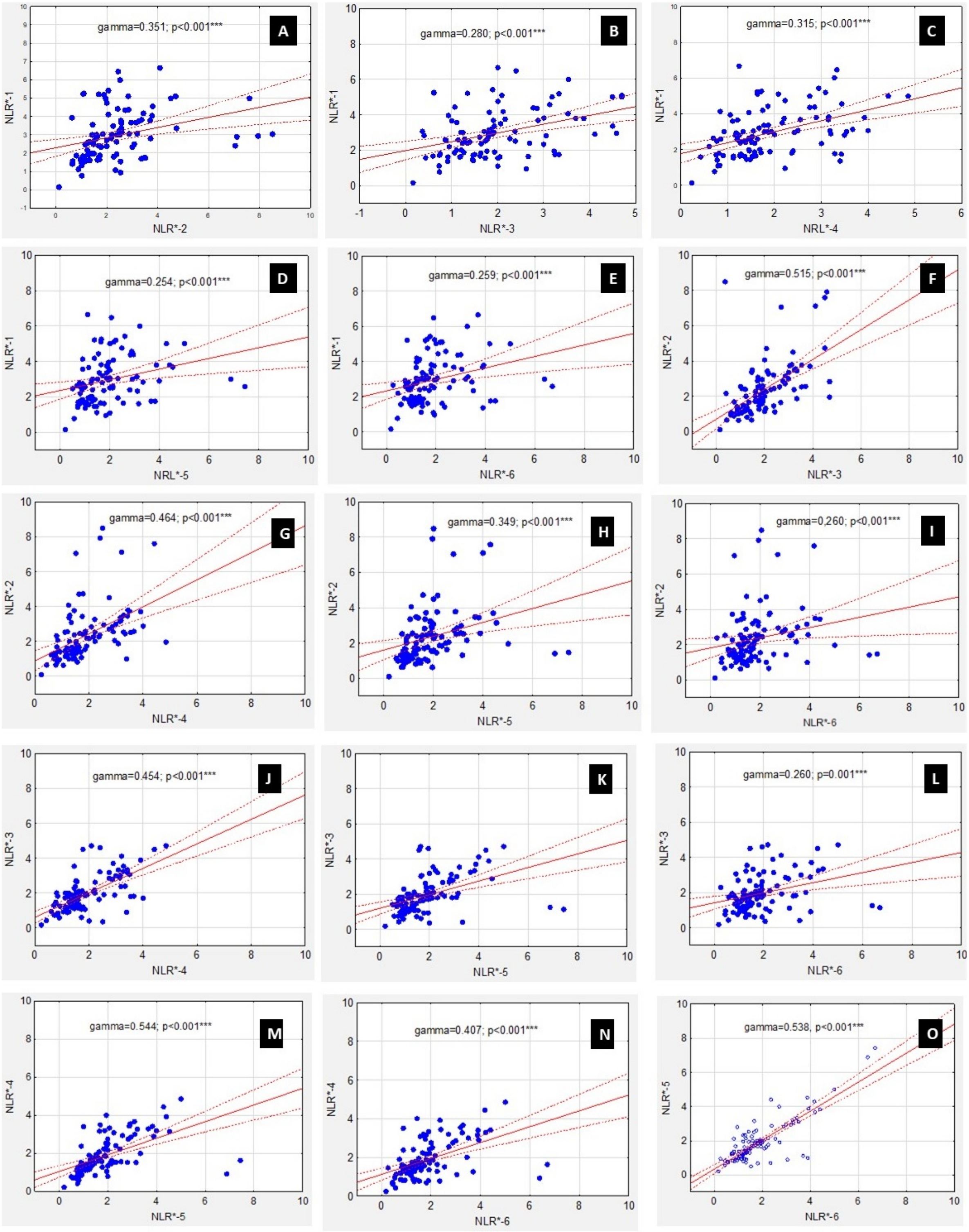

Figure S1. (**A–O**) Correlation between the neutrophil-to-lymphocyte ratios during first-line chemotherapy in ovarian cancer patients. NLR*—neutrophil-to-lymphocyte ratio; *** *p* statistically significant.

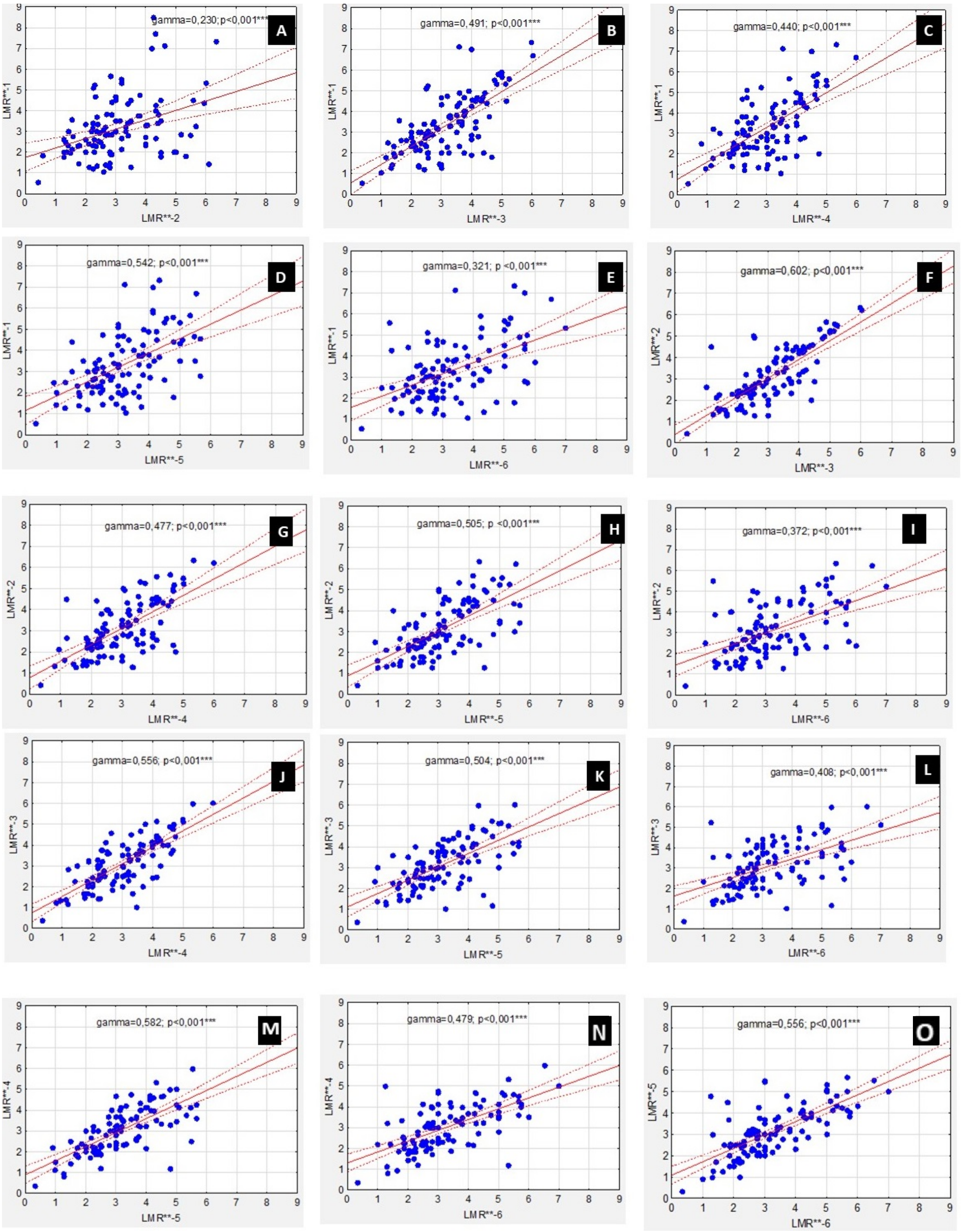

Figure S2. (**A–O**) Correlation between lymphocyte-to-monocyte ratios during first-line chemotherapy in ovarian cancer patients. LMR**—lymphocyte-to-monocyte ratio; *** *p* statistically significant.

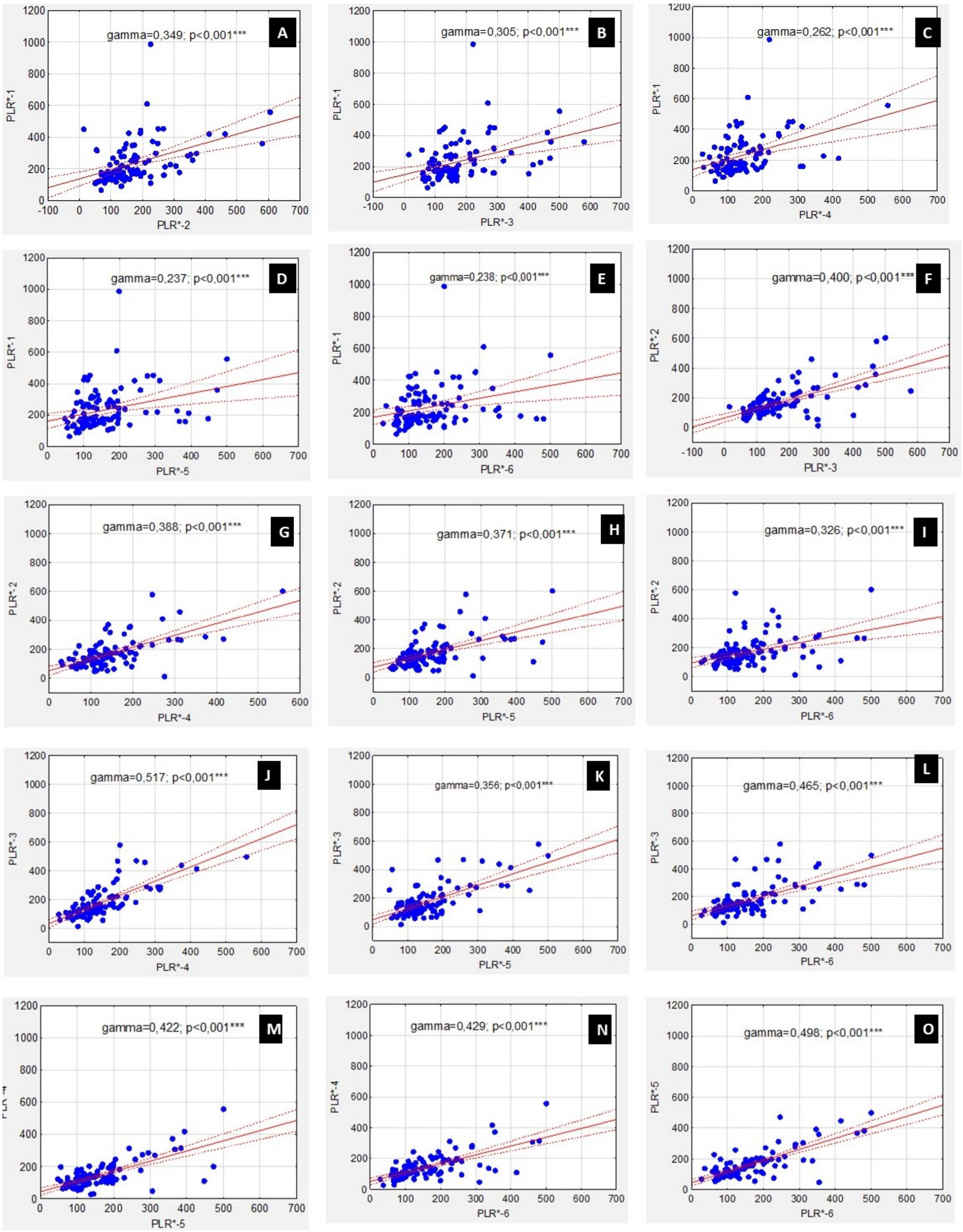

Figure S3. (**A–O**) Correlation between the platelet-to lymphocyte ratios during first-line chemotherapy in ovarian cancer patients. PLR*—lymphocyte-to-monocyte ratio; *** *p* statistically significant.

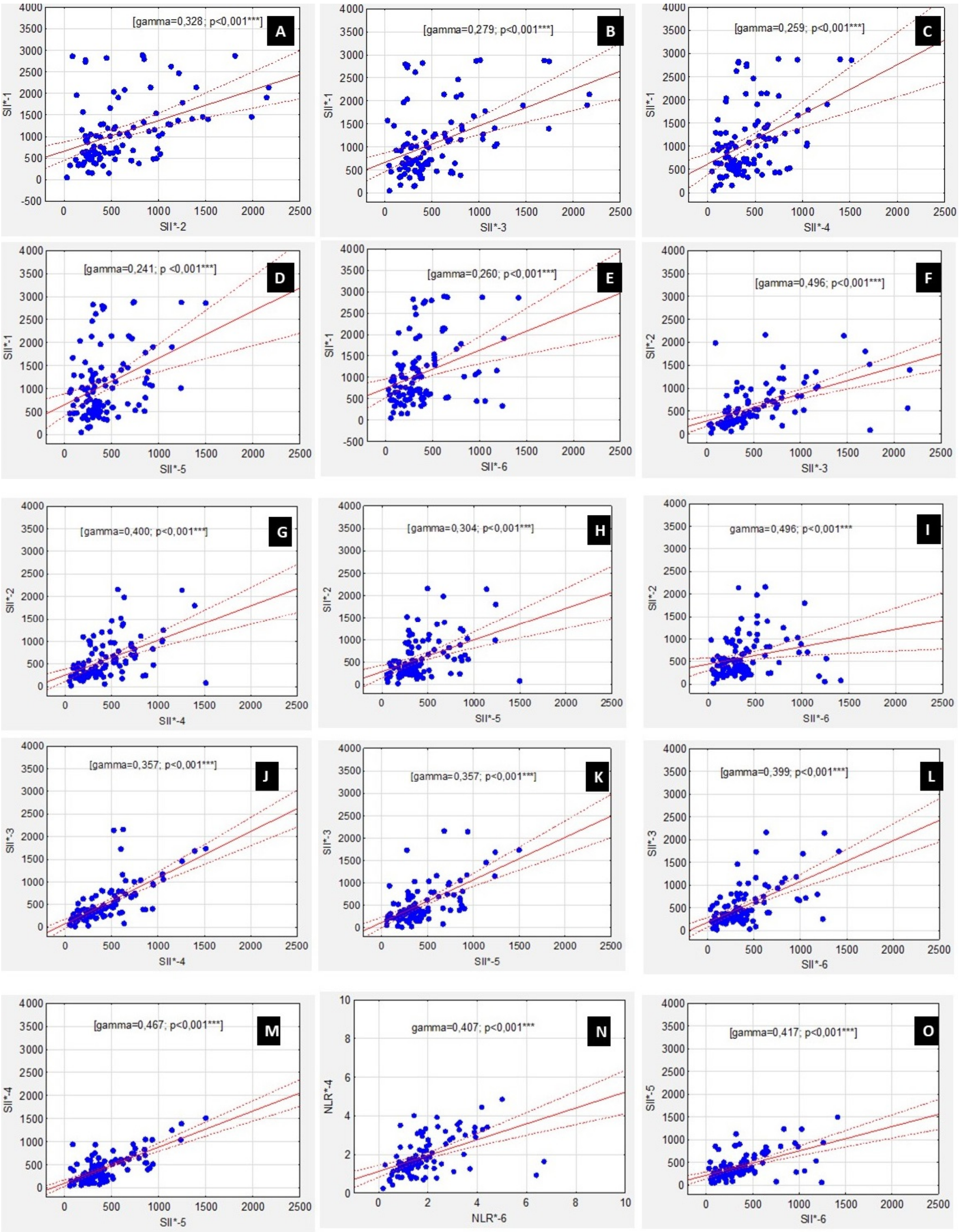

Figure S4. (**A–O**) Correlation between the systemic inflammatory index values during first-line chemotherapy in ovarian cancer patients. SII*—systemic inflammatory index; *** *p* statistically significant
